# Optimizing Antibacterial Essential Oil Blends from *Helichrysum plicatum*, *Petroselinum crispum*, and *Origanum vulgare* for Dairy Preservation: Mixture Design and In Silico Analysis

**DOI:** 10.3390/foods15040675

**Published:** 2026-02-12

**Authors:** Jelena Živković, Jovana Petrović, Mohamed El Fadili, Dejan Pljevljakušić, Sara Lebrazi, Dejan Stojković, Katarina Šavikin, Mouhcine Fadil

**Affiliations:** 1 Institute for Medicinal Plants Research “Dr. Josif Pančić”, Tadeuša Košćuška 1, 11000 Belgrade, Serbia; dpljevljakusic@mocbilja.rs (D.P.); ksavikin@mocbilja.rs (K.Š.); 2 Institute for Biological Research “Siniša Stanković”—National Institute of the Republic of Serbia, University of Belgrade, Bulevar Despota Stefana 142, 11108 Belgrade, Serbia; jovana0303@ibiss.bg.ac.rs (J.P.); dejanbio@ibiss.bg.ac.rs (D.S.); 3 LIMAS Laboratory, Faculty of Sciences Dhar El Mehraz, Sidi Mohammed Ben Abdellah University, P.O. Box 1796 Atlas, Fez 30000, Morocco; mohamed.elfadili@usmba.ac.ma; 4 Microbial Biotechnology and Bioactive Molecules Laboratory, Faculty of Sciences and Techniques, Sidi Mohamed Ben Abdellah University, Road of Imouzzer, P.O. Box 2202, Fez 30000, Morocco; sara.lebrazi@usmba.ac.ma; 5 Laboratory of Applied Organic Chemistry, Faculty of Sciences and Techniques, Sidi Mohamed Ben Abdellah University, Road of Imouzzer, P.O. Box 2202, Fez 30000, Morocco; mouhcine.fadil@usmba.ac.ma

**Keywords:** milk, *Listeria monocytogenes*, *Escherichia coli*, mixture design, in silico

## Abstract

This study demonstrates the potential of *Helicrisum plicatum* (*H. plicatum*), *Petroselinum crispum* (*P. crispum*) and *Origanum vulgare* (*O. vulgare*) essential oils and their combination against four strains of two bacterial species, *Listeria monocytogenes* (*L. monocytogenes*) and *Escherichia coli* (*E. coli*), isolated from milk. GC/MS and GC/FID analyses of *H. plicatum* essential oil revealed α-pinene (27.61%), γ-curcumene (20.7%) and neryl acetate (5.88%) as the main compounds present in *H plicatum* essential oil. The main components of the essential oil of *P. crispum* were α-pinene (17.34%), 1,3,8-*p*-menthatriene (23.66%), β-phellandrene (10.41%) and myristicin (12.7%). In *O. vulgare* essential oil, carvacrol (58.3%), *o*-cymene (15.4%) and thymol (6.0%) were predominant compounds. Subsequently, an augmented simplex-centroid mixture design was applied to optimize the antibacterial efficacy of EO formulations against *L. monocytogenes* and *E. coli*. The simultaneous optimization all responses indicates that the optimal antimicrobial formulation was achieved using a ternary mixture of *H. plicatum*, *P. crispum*, and *O. vulgare* in a 16:16:68 (*v*/*v*/*v*) ratio. In parallel, an in silico study of four main essential oil components evaluated their drug-likeness, pharmacokinetic and toxicity properties, and binding to bacterial targets. These major compounds satisfy the main standards for drug-like molecules, with simulations pointing to good oral absorption, an acceptable safety profile, and stable binding to key enzymes in *E. coli* and *L. monocytogenes*, which supports their antibacterial potential. Overall, these combined experimental and computational results point to oregano, parsley, and immortelle essential oils as promising natural antimicrobial options for improving the preservation of dairy products.

## 1. Introduction

Despite significant advancements in health control measures within the food production industry, data from the World Health Organization (WHO) indicate that approximately 600 million people globally are affected by foodborne diseases (FBDs), leading to an estimated 420,000 deaths annually [1]. Dairy products contribute to around 14% of these cases. This is largely due to the potential presence of harmful pathogens in dairy, including *L. monocytogenes*, *Salmonella* spp., *Staphylococcus aureus*, and *E. coli*. Frequent outbreaks caused by *L. monocytogenes* and *E. coli* O157:H7 in milk and dairy products highlight the urgent need for safer and more natural preservation strategies. *L. monocytogenes* is particularly concerning due to its remarkable ability to withstand a wide range of physico-chemical conditions, including refrigeration temperatures, acidic environments, and high salt concentrations [2]. El Otmani et al., reported the presence of *L. monocytogenes* ATCC 15313 in UHT milk [3], while *E. coli* O157:H7 has been directly associated with several dairy-related outbreaks [4]. In light of these risks, identifying effective natural antimicrobial agents, such as essential oils, offers a promising alternative to synthetic preservatives in ensuring microbiological safety in dairy products [5].

The dairy sector has placed a strong emphasis on maintaining microbiological quality to ensure food safety and mitigate potential economic losses. As a result, there has been an increased reliance on preservatives with antimicrobial properties in food formulations [6].

Also, use of artificial preservatives such as sorbate, sulfite, and nitrite has been linked to serious health risks, including hypersensitivity, asthma, neurological damage, hyperactivity, and cancer. Consequently, consumers increasingly prefer natural food preservatives over synthetic alternatives, perceiving them as safer options [6]. In this context, natural antimicrobial compounds offer a promising alternative for preserving and extending the shelf life of food products [1].

Extensive research has been conducted on essential oils (EOs) to investigate their pharmacological effects, which cover a broad spectrum of applications. The versatility of these oils, along with their diverse benefits, makes them valuable compounds with potential uses in fields such as medicine, pest control, and beyond [7]. They have been reported to possess antibacterial properties against a wide range of pathogenic bacteria. Due to these medicinal properties, they are commonly used as preservatives in dairy products and other stored foods [5]. The combination of different essential oils (EOs) could enhance their preservative effects, reducing their sensory impact on food by making them effective even at lower concentrations [8]. Employing mixture design model proves to be an effective approach in reducing the number of experimental trials, while simultaneously enabling the identification of optimal responses and ideal formulations through the application of advanced mathematical and statistical models [9].

Hence, in this work we attempted to ascertain whether it is possible to approximate the most effective antimicrobial blend of three essential oils using a simplex-lattice mixture design. The antimicrobial activity of the blends was evaluated against *L. monocytogenes* and *E. coli* strains. The selected essential oils were used as a model system of components exhibiting higher and lower antimicrobial activity in order to design the most effective antimicrobial combination. All studied essential oils are generally recognized as safe and have been used as flavoring ingredients in the food industry.

## 2. Materials and Methods

### 2.1. Essential Oils Sources

Essential oils (EOs) were commercially sourced from two different retailers. Immortelle (*Helichrysum italicum*) EO was obtained from Herba d.o.o. (Belgrade, Serbia), while parsley (*P. crispum*) EO and Greek oregano (*Origanum heracleoticum*) EOs were purchased from Pharmanais d.o.o. (Babušnica, Serbia).

### 2.2. GC and GC-MS Analysis

GC analyses were performed without experimental replicates due to the use of commercially available essential oils assumed to be homogeneous mixtures. Essential oil samples were prepared by dissolving 20 µL of essential oil in 1.8 mL of GC-grade technical ethanol prior to analysis and subsequently analyzed using gas chromatography (GC) and gas chromatography–mass spectrometry (GC-MS). The analyses were performed on a Shimadzu GC-MS-QP2010 Ultra mass spectrometer equipped with a flame ionization detector (FID) and coupled to a GC-2010 gas chromatograph. Separation was carried out using an InertCap5 capillary column (60.0 m × 0.25 mm × 0.25 µm). Helium (He) was used as the carrier gas, with a split ratio of 1:5 and a linear velocity of 35.2 cm/s. The oven temperature was initially set at 60 °C and maintained for 4 min, then increased to 280 °C at a rate of 4 °C/min and held for 10 min. The injector and detector temperatures were set at 250 °C and 300 °C, respectively, while the ion source temperature was maintained at 200 °C. Compound identification was performed using a two-dimensional approach that combined comparison of experimental retention indices calculated relative to a homologous series of n-alkanes (C7–C40) with mass spectral matching. Mass spectral deconvolution and identification were conducted using the AMDIS software (version 2.71) employing probability-based matching (PBM) algorithms [10], with spectral comparisons to the NIST 14 [11] and Wiley 11 [12], mass spectral libraries performed using the Shimadzu Postrun software (version 4.11), and literature retention indices obtained from Adams [13].

### 2.3. Experimental Design

#### 2.3.1. Augmented Simplex-Centroid Design

**Figure 1:**
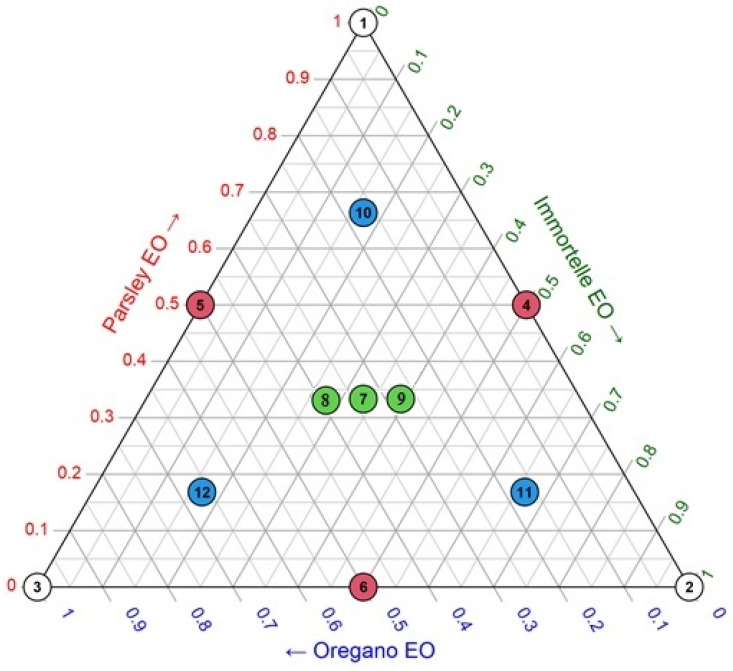
An illustration of augmented simplex-centroid design for three-component mixture. White points (Runs 1–3): Pure components (vertices), Red points (Runs 4–6): Binary edge mixtures, Green points (Runs 7–9): Ternary equal proportions mixtures, Blue points (Runs 10–12): Augmented (axial) mixtures.

In this study, an augmented simplex-centroid design was employed to determine the optimal antimicrobial activity of a combination of essential oils from *P. crispum*, *Helichrysum plicatum*, and *O. vulgare*. A total of 12 experiments were performed, and are represented as an equilateral triangle (Figure 1). The triangle’s three corners (X1, X2, X3) represent the three pure components (1). The midpoints of the sides (X4, X5, X6) represent binary mixtures (0.5/0.5), while the center of the triangle (X7) represents a mixture with equal proportions of all three components (0.33/0.33/0.33). Each trial was repeated three times. Additionally, the three control points (X10, X11, X12) correspond to ternary mixtures with the proportions 0.67/0.16/0.16. Augmented axial points were positioned at the midpoint between the centroid and each binary blend along vertex-centroid axes to enhance estimation of cubic term coefficients [14]. The experiment involving the equal-proportion mixture was repeated three times to assess the model’s lack of fit. In total, twelve experimental runs were conducted as part of the current exploratory design.

The mixture components satisfied the standard unity constraint, Σ*xᵢ* = 1 (Equation (1)) [15].

(1) $$\sum_{i=1}^{n}x_{i}=100\%$$

#### 2.3.2. Mathematical Postulated Model

To capture the complex linear interactions of the three essential oils, several models, including linear (Equation (2)), quadratic (Equation (3)) and special cubic (Equation (4)) were tested [16].

(2) $$Y_{linear}={\alpha_{1}X}_{1}+{\alpha_{2}X}_{2}+\alpha_{3}X_{3}+ɛ$$

(3) $$Y_{quadratic}={\alpha_{1}X}_{1}+{\alpha_{2}X}_{2}+{\alpha_{3}X}_{3}+\alpha_{12}X_{1}X_{2}+\alpha_{13}X_{1}X_{3}+\alpha_{23}X_{2}X_{3}+ɛ$$

(4) $$Y_{Special\;cubique}={\alpha_{1}X}_{1}+{\alpha_{2}X}_{2}+{\alpha_{3}X}_{3}+\alpha_{12}X_{1}X_{2}+\alpha_{13}X_{1}X_{3}+\alpha_{23}X_{2}X_{3}+\alpha_{123}X_{1}X_{2}X_{3}+ɛ$$

Model selection was based on validation parameters, specifically the overall model significance (ANOVA *p*-value), the coefficient of determination (R^2^), adjusted R^2^, predicted R^2^, and the lack-of-fit *p*-value. The comparative analysis revealed that the linear and quadratic models were insufficient, presenting notably high ANOVA *p*-value, lower R^2^ values and significant lack-of-fit (*p* < 0.05) compared to the special cubic model. Consequently, the special cubic model was selected as it demonstrated the highest goodness-of-fit and adjusted R^2^, confirming it as the most statistically robust model for this study.

The response vector Y comprises MIC (mg/mL); βᵢ, βᵢⱼ, and β_123_ denote linear, binary, and ternary interaction coefficients, respectively, and ε is the random error. For all responses, we fitted our data to a special cubic model. This chosen model-comprising linear, binary, and ternary interaction terms-was selected because it alone can reveal three-component synergy or antagonism, and it yielded the highest R^2^ and R^2^adj among the candidate models.

#### 2.3.3. Statistical Analysis

The models were validated using a combination of statistical tools to ensure accuracy, reliability, and predictive power. We employed ANOVA to test the models that were well adjusted to the data. The F-Ratio is defined as the ratio of regression mean squares to residual mean squares; this was the basis of the evaluation. This ratio was then tested against the theoretical F ratio for the respective degrees of freedom. The coefficients of determination, R^2^ and R^2^adj, were used to measure the fitness of the models constructed. The statistical significance of calculated coefficients was assessed by the Student’s *t*-test. Furthermore, the models were validated using test points selected based on the optimum conditions identified during the optimization process. JMP^®^ software v.16 and Expert Design software v.12 was used for experimental design, statistical analysis, and graphical representation. The results are expressed as mean ± standard deviation (SD). Statistical significance was considered for *p*-value < 0.05 [17].

### 2.4. Optimization Tools

Two- and three-dimensional contour diagrams were generated to map the dependence of each measured response on the essential-oil proportions. Isoresponse lines connect mixture compositions yielding identical predicted values; warm-coloured regions (red) indicate maxima, whereas cool-colored regions (blue) denote minima. Inspection of contour curvature allowed rapid identification of synergistic zones and feasible compromise regions where several responses approach their extrema simultaneously. Optimum formulations were located with the desirability function approach. Each response yᵢ was converted into an individual desirability index d_i_ [0, 1] through a one-sided or two-sided transformation, where dᵢ = 0 corresponds to a completely unacceptable value and dᵢ = 1 to the ideal target [18].

### 2.5. Antimicrobial Activity

The following Gram-positive bacteria were tested: *L. monocytogenes* (ATCC 15313), *L. monocytogenes* (ATCC 19111), *L. monocytogenes* (ATCC 13932), as well as Gram-negative bacteria *E. coli* O157:H7 ATCC 43888 and *E. coli* O157:H7 (ATCC 700728). Microorganisms are deposited at the Mycological Laboratory, Department of Plant Physiology, Institute for Biological Research “Siniša Stanković,” National Institute of the Republic of Serbia, University of Belgrade.

Antibacterial activity was evaluated using the microdilution method previously described by Soković et al. [19]. Cultures of microorganisms were cultivated overnight at 37 °C in Tryptic Soy Broth (TSB) and adjusted to a density of 1.0 × 10^5^ CFU/mL with sterile saline. Test samples dissolved in 30% ethanol were added to 100 μL of TSB medium, along with 1.0 × 10^4^ CFU per well of bacterial inoculum. After overnight incubation at 37 °C, 40 μL of p-iodonitrotetrazolium chloride solution (0.2 mg/mL) was added to each well, and plates were incubated for an additional 60 min at 37 °C to allow color development. The minimal inhibitory concentration (MIC) was determined as the lowest concentration that caused a visible reduction in color intensity (light red compared to deep red in untreated controls) or complete absence of color. Minimal bactericidal concentration (MBC) was determined by serial sub-cultivation, transferring 2 μL from each well to fresh broth and incubating for 24 h at 37 °C. MBC was defined as the lowest concentration that completely eradicated bacterial growth, corresponding to a 99.5% reduction of the initial inoculum. Commercial preservative E211 was used as a positive control.

### 2.6. In Silico Screening

In the present study, four main compounds M α-pinene (M1), γ-curcumene (M2), 1,3,8-*p*-menthatriene (M3) and carvacrol (M4) obtained from *P. crispum*, *H. plicatum* and *O. vulgare*, were subjected to an integrated computational investigation, including the prediction of physicochemical and pharmacokinetic profiles [20,21], boiled-egg application [22,23], bioavailability radars [24,25], in addition to molecular docking simulations towards two receptor proteins: the crystal structure of *E. coli* DNA Gyrase B, in complex with 4-(4-bromo-1H-pyrazol-1-yl)-6-[(ethylcarbamoyl)amino]-N-(pyridin-3-yl)pyridine-3-carboxamide (PDB ID of 6F86), and the crystal structure of penicillin-binding protein from *L. monocytogenes* (PDB ID of 5ZQB), targeting *E. coli* and *L. monocytogenes* pathogenic strains, respectively. Both targeted proteins were prepared in a standardized manner, removing all co-crystallized ligands [26,27], adding polar hydrogens and Gasteiger charges [28,29,30], and then docked to four main compounds: α-pinene, γ-curcumene, 1,3,8-p-menthatriene, and carvacrol, by using Auto Dock software (V. 4.2) [31,32,33], where the produced interactions were visualized in two- and three-dimensions using Discovery-Studio software (V. 2021) [34,35].

## 3. Results

### 3.1. Essential Oils Chemical Analysis

The results of chemical analyses of essential oils from immortelle, parsley, and Greek oregano are presented in Table 1, where 77, 51, and 50 compounds were identified, respectively, with over 99% of the constituents identified in all cases. In the essential oil of immortelle, the total content of monoterpenoids and sesquiterpenoids was relatively balanced (43.1% and 50.6%, respectively). In contrast, the content of monoterpenoid compounds in the oils of parsley and oregano was significantly higher (76.9% and 94.4%, respectively).

The most dominant monoterpenoid compounds in the essential oil of immortelle were α-pinene, neryl acetate, and sylvestrene (27.6%, 5.9%, and 3.3%, respectively), while the sesquiterpene fraction was dominated by γ-curcumene, β-bisabolene, aromadendrene, and 7-epi-α-selinene (20.7%, 6.1%, 4.9%, and 4.0%, respectively). Among β-diketones, which are of particular importance when assessing the quality of immortelle essential oil, only one compound with a molecular mass of 224 *m*/*z* was identified in a concentration of 1.2%. This corresponds to 2,4,6,9-tetramethyldec-8-ene-3,5-dione, which we referred to in the table under the retention index 1495.8 by its colloquial name, italidione II. However, its isomer with the same molecular mass, as well as italidione I (MW = 210) and italidione III (MW = 238), were not identified in this oil. The chemical composition of the analyzed immortelle essential oil aligns with previously reported chemical profiles of this oil from the France and Bosnia [36]. It has long been observed that the chemical composition of immortelle essential oils from the former Yugoslav region differs significantly from the popular Corsican oil [37]. Immortelle from Corsica produces an oil rich in neryl acetate and β-diketones (commonly referred to as “italidiones”), while oils from Croatia, Serbia, and Bosnia and Herzegovina are predominantly characterized by α-pinene, γ-curcumene, and β-selinene, with relatively lower levels of neryl acetate and italidiones [38,39,40].

The dominant monoterpenoid fraction of parsley essential oil was exclusively represented by non-oxygenated monoterpenes, such as 1,3,8-p-menthatriene, α-pinene, β-pinene, β-phellandrene, myrcene, and terpinolene (23.7%, 17.3%, 12.4%, 10.4%, 5.1%, and 4.6%, respectively). In addition to the monoterpene fraction, this essential oil also contained a significant proportion of phenylpropanoid compounds (21.9%), with the most abundant representatives being myristicin (12.7%), 1-allyl-2,3,4,5-tetramethoxybenzene (4.2%), and elemicin (3.4%). The fractions of oxygenated mono- and sesquiterpenes, as well as non-oxygenated sesquiterpenes, did not exceed 1% in any case. The composition of the identified dominant compounds in the analyzed sample of parsley essential oil largely corresponds to previously published chemical analyses of this oil [41]. Particularly noteworthy is the phenylpropanoid fraction, which constitutes approximately one-fifth of the oil in our sample and is of great interest due to its potent biological activities [42,43].

The essential oil of Greek oregano is composed almost exclusively of monoterpenes (94.4%), with the non-oxygenated fraction accounting for 27.0% and the oxygenated fraction contributing 67.4%. The main representatives of the non-oxygenated monoterpenes in this oil are o-cymene (15.4%) and γ-terpinene (5.1%). Meanwhile, the majority of the oxygenated monoterpene fraction consists of the phenolic compounds carvacrol and thymol (58.3% and 6.0%, respectively). The sesquiterpene fraction in the Greek oregano essential oil is modestly represented, making up only 3.3%. Consistent with our findings, most reported chromatographic analyses of Greek oregano essential oil highlight carvacrol as the dominant phenolic compound, accounting for more than half of the oil [44,45]. Due to the remarkable antimicrobial and antioxidant properties of the phenolic compounds present in this oil, it has been extensively studied for its biological activities [46,47].

### 3.2. Antibacterial Activity of Individual Essential Oils

The antibacterial activity depends on both the specific properties of the essential oil and the susceptibility of the tested microbial species. The MIC values of three essential oils against the four microbial strains reveal distinct patterns of antimicrobial activity (Table 2). Among the pure essential oils evaluated (experiments 1–3), *O. vulgare* showed the strongest antimicrobial effect. *Helichrysum plicatum* and *P. crispum* demonstrated similar activity against *L. monocytogenes* strains; however, *H. plicatum* showed stronger inhibitory effects against *E. coli* strains. Considering the essential oil of *O. vulgare*, its effect was notably more potent than that of the tested preservative, sodium benzoate (E211).

Few reports on antimicrobial activity of *O. vulgare* are available in the literature. Santoyo et al., investigated the antimicrobial activity of oregano essential oil extracted via supercritical fluid extraction (SFE) against various bacterial strains, including *E. coli* ATCC 11775, and found that the fraction enriched in carvacrol exhibited the highest antimicrobial efficacy [48]. Govaris et al. have demonstrated that the incorporation of oregano and thyme essential oils into feta cheese significantly reduces the survival of *Escherichia coli O157:H7* and *Listeria monocytogenes* during refrigerated storage under modified atmosphere packaging [49]. The antibacterial effect was dose-dependent, with higher concentrations of oregano essential oil leading to a more pronounced reduction in pathogen survival time. The observed activity was attributed to the high content of phenolic compounds, particularly carvacrol and thymol, which are known to disrupt bacterial cell membranes. In a study by Linde et al., parsley essential oil demonstrated inhibitory activity against all tested bacterial strains, with minimum inhibitory concentrations (MICs) ranging from 0.04 to 1.00 mg/mL [50]. Furthermore, it exhibited bactericidal effects, with minimum bactericidal concentrations (MBCs) ranging from 0.15 to 10.00 mg/mL. Among the tested strains, *E. coli* was the most resistant, with an MBC value of 10.00 mg/mL. To date, there are no published studies reporting the antibacterial activity of *H. plicatum* essential oil against major foodborne pathogens such as *L. monocytogenes* and *E. coli*. However, previous research has shown that ethanolic extracts of *H. plicatum* exhibit inhibitory effects against *E. coli* O157:H7, suggesting that bioactive constituents of this plant may be useful in the ongoing challenge of controlling the growth of pathogenic *E. coli* [51]. These findings indicate that *H. plicatum* represents an underexplored source of antimicrobial compounds and support further investigation of its essential oil as a potential antibacterial agent.

Moreover, numerous studies have demonstrated that the antimicrobial effects of essential oils are often the result of a synergistic interaction between their primary and secondary constituents. Additionally, it has been shown that volatile compounds are capable of associating with bacterial cells and penetrating their phospholipid membranes, which ultimately leads to bacterial cell death [52].

Cusimano et al. evaluated the antimicrobial properties of thymol—a principal monoterpenoid component of Greek oregano essential oil—against planktonic cells and biofilm formation in bacterial isolates [53]. Their study included 25 *L. monocytogenes* strains derived from various food sources and five *E. coli* strains collected from a farm environment. Minimum inhibitory concentration (MIC) testing revealed that thymol inhibited *L. monocytogenes* at concentrations between 250 and 400 μg/mL, while the *E. coli* isolates, characterized by virulence and resistance traits, exhibited MIC values ranging from 300 to 400 μg/mL.

### 3.3. Antibacterial Activity of Combinations of Essential Oils

The next step was to obtain a mixture of these oils with the greatest ability to inhibit the growth of microorganisms. For this purpose, an augmented simplex-lattice design was developed (Table 2), and the resulting essential oil mixtures were evaluated for their antimicrobial activity against *E. coli* ATCC 11775, *E. coli* O157:H7 (ATCC 700728), and two strains of *L. monocytogenes* (ATCC 15313 and ATCC 19111). To the best of our knowledge, no previous studies have investigated the combined effect of essential oils from *O. vulgare*, *H. plicatum*, and *P. crispum* using experimental design methodology. This novel approach represents a promising and innovative direction in the optimization of natural antimicrobial agents.

### 3.4. Statistical Validation of the Postulated Model

**Figure 2:**
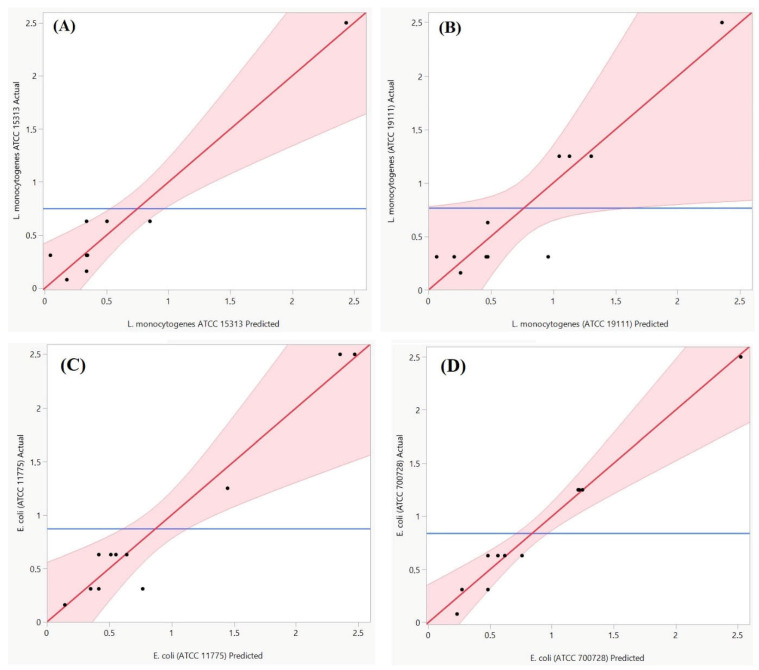
Curves of actual response in terms of predicted ones for IC LM1 (**A**), MIC LM2 (**B**), MIC EC1 (**C**), and MIC EC2 (**D**). The shaded bands indicate the model confidence limits.

The experimentally obtained MIC data were statistically analyzed and fitted to various response surface models to explore the relationship between the antimicrobial response and influencing factors for each bacterial strain. Special cubic model evaluation was conducted using analysis of variance (ANOVA). Model adequacy was determined based on statistical significance (*p* < 0.05) and the adjusted coefficient of determination (R^2^) (Table 3). The main effect of the regression is significant since the probability of risk significance *p*-value is less than 0.05. In addition, the test for lack of fit, the selected models do not show a lack of fit, since the significance of the *p*-value risk is greater than 0.05 for all five responses. Moreover, the coefficients of determination obtained 0.96, 0.87, 0.94, and 0.97 for MIC LM1, MIC LM2, MIC EC1, and MIC EC2, respectively, showed a good agreement between the experimental and predicted mathematical models as shown in Figure 2.

### 3.5. Essential Oil Effects and Fitted Model

Table 4 summarizes the estimated regression coefficients of the special model. Statistically significant coefficients (*p* < 0.05) were used to determine the relationships between the investigated factors and the observed responses for MIC LM1, MIC LM2, MIC EC1, and MIC EC2.

The statistical analysis identified significant coefficients (*p* < 0.05) for each bacterial strain, revealing distinct patterns of individual and interactive effects among Immortelle, Parsley, and Oregano essential oils. The resulting mathematical models demonstrate how these effects translate to antimicrobial activity, measured by changes in minimum inhibitory concentration (MIC).

#### 3.5.1. Antimicrobial Effect on *L. monocytogenes* (ATCC 15313)

For this strain, the interactions between the oils are predominantly synergistic, leading to a significant enhancement of their antimicrobial properties. The mathematical model that describes the Minimum Inhibitory Concentration (MIC) is:

Y*_L. monocytogenes_*_ATCC 15313_ = 2.436 × X_1_ + 2.436 × X_2_ − 7.735 × X_1_X_2_ − 3.851 × X_1_X_3_ − 3.851 × X_2_X_3_ + ɛ

The positive linear coefficients for Immortelle (b_1_ = 2.436) and Parsley (b_2_ = 2.436) indicate that when used alone, these oils have limited efficacy. However, the large, negative coefficients for all binary interaction terms signify strong synergistic effects. The most potent synergy is observed between Immortelle and Parsley (b_12_ = −7.735), which dramatically reduces the MIC. The combinations of Immortelle with Oregano (b_13_ = −3.851) and Parsley with Oregano (b_23_ = −3.851) also demonstrate significant synergism. This means that combining these oils creates a formulation far more potent than the sum of its parts, making it highly effective against this particular strain.

#### 3.5.2. Antimicrobial Effect on *L. monocytogenes* (ATCC 19111)

In stark contrast to the previous strain, the interaction between Immortelle and Parsley against *L. monocytogenes* (ATCC 19111) is antagonistic. The governing model is:

Y*_L. monocytogenes_*_ATCC 19111_ = 1.3056 × X_1_ + 1.0492 × X_2_ + 4.7096 × X_1_X_2_ + ɛ

Here, while Immortelle and Parsley individually show some weak antimicrobial activity (positive linear coefficients), their combination is detrimental to the overall effect. The statistically significant and positive interaction coefficient (b_12_ = 4.7096) indicates antagonism, meaning the oils interfere with each other’s mechanisms of action. This leads to a higher MIC value than would be expected, rendering the mixture less effective than its individual components against this specific strain.

#### 3.5.3. Antimicrobial Effect on *E. coli* (ATCC 11775)

The activity against this *E. coli* strain is uniquely characterized by a powerful three-way synergistic interaction. The predictive model is:

Y*_E.coli_*_ATCC 11775_ = 2.4692 × X_1_ − 21.312 × X_1_X_2_X_3_ + ɛ

Individually, only Immortelle shows a statistically significant (but weak) effect. The binary interactions are not significant. However, the ternary interaction term (b_123_) has a very large and negative coefficient (−21.312), indicating a profound synergistic effect when all three oils—Immortelle, Parsley, and Oregano—are present together. This highlights a complex interaction where the presence of all three components is required to unlock a potent antimicrobial activity that is entirely absent in the pairwise combinations.

#### 3.5.4. Antimicrobial Effect on *E. coli* (ATCC 700728)

Against this final strain, the antimicrobial action is driven by significant binary synergies, particularly involving Immortelle. The model is defined as:

Y*_E.coli_*_ATCC 700728_ = 2.5262 × X_1_ + 1.2207 × X_2_ − 2.5063 × X_1_X_2_ − 3.1226 × X_1_X_3_ + ɛ

As seen with other strains, Immortelle and Parsley have weak individual effects. The key finding here is the significant synergistic interactions shown by the negative coefficients for ImmortelleParsley (b_12_ = −2.5063) and ImmortelleOregano (b_13_ = −3.1226). Both combinations enhance the overall antimicrobial potency, with the synergy between Immortelle and Oregano being slightly more pronounced. This demonstrates that for this strain, combining Immortelle with either Parsley or Oregano is an effective strategy to boost antimicrobial performance.

### 3.6. Optimization of EO Mixtures and Desirability Analysis

Through MDM optimization, the objective is to identify the essential oil formulation that produces optimal results for the evaluated responses. The target mixture should achieve peak antimicrobial effectiveness, indicated by minimal MIC values.

**Figure 3:**
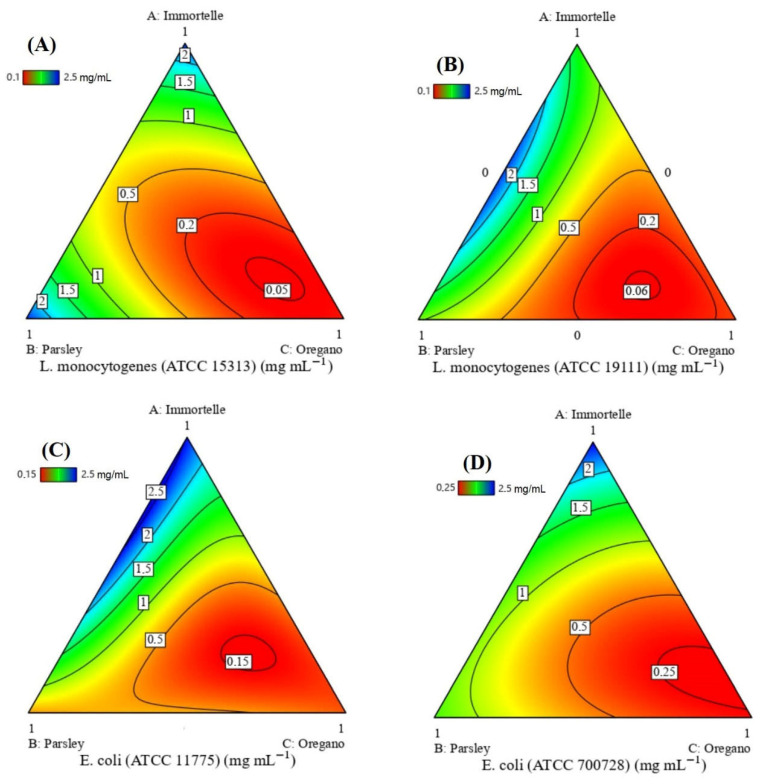
Contour plots of the compromise area for the optimal MIC LM1 (**A**), MIC LM2 (**B**), MIC EC1 (**C**) and MIC EC2 (**D**).

**Figure 4:**
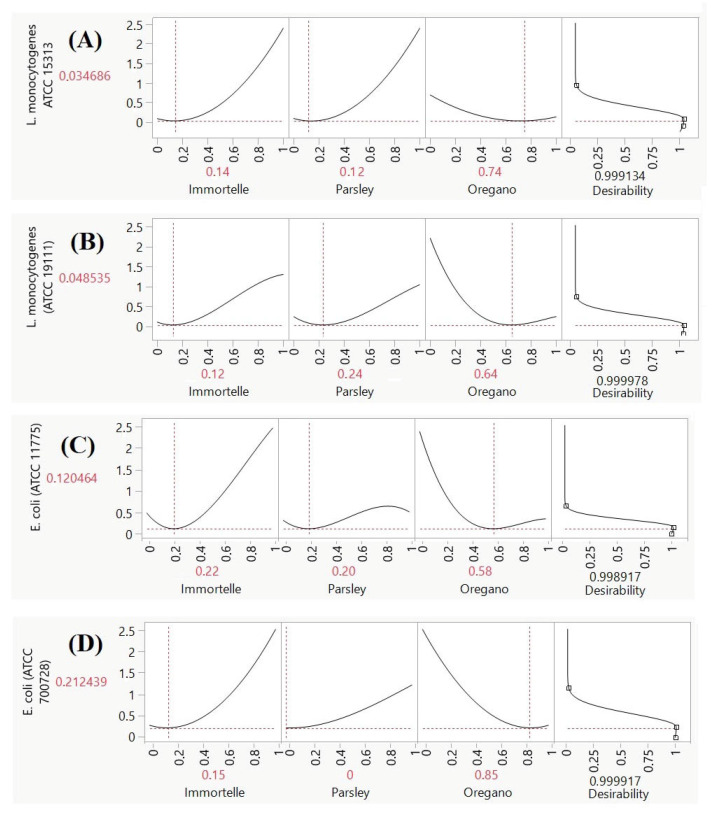
Desirability plots of the optimum value for MIC LM1 (**A**), MIC LM2 (**B**), MIC EC1 (**C**) and MIC EC2 (**D**).

The contour plot analysis (Figure 3A) indicates that a MIC of approximately 0.05 mg/mL against *L. monocytogenes* (ATCC 15313) is achievable with a ternary mixture of the essential oils, particularly with formulations rich in Oregano. To identify the ideal blend, a desirability analysis was performed as shown in Figure 4A, which predicted an optimal MIC of 0.034 mg/mL. This optimal response, corresponding to a high desirability score of 0.99, is achieved with a specific volumetric ratio of Immortelle, Parsley, and Oregano at 14:12:74 (*v*/*v*/*v*).

Similarly, for *L. monocytogenes* (ATCC 19111), the contour plot (Figure 3B) suggests a MIC of approximately 0.06 mg/mL is attainable. However, the desirability analysis identified a more potent optimal formulation, predicting a MIC of 0.048 mg/mL. This optimal response is also associated with a desirability score of 0.99 and is obtained using a ternary mixture of Immortelle, Parsley, and Oregano in a 12:24:64 (*v*/*v*/*v*) ratio (Figure 4B).

For the *E. coli* (ATCC 11775) strain, analysis of the contour plot (Figure 3C) reveals that the optimal zone for antimicrobial activity lies within the ternary mixture of all three essential oils, corresponding to a MIC of approximately 0.15 mg/mL. To further refine these conditions, a desirability analysis was performed, which predicted an optimal MIC of 0.12 mg/mL. Figure 4C shows that this peak response, associated with a high desirability score of 0.99, is achieved with a specific formulation of Immortelle, Parsley, and Oregano in a volumetric ratio of approximately 22:20:58 (*v*/*v*/*v*).

Regarding the antimicrobial activity against *E. coli* (ATCC 700728), the analysis revealed that the optimal effect is achieved with a binary combination of Immortelle and Oregano essential oils (Figure 3D). The contour plot analysis indicates that the optimal zone exists within this binary region, corresponding to a MIC of approximately 0.25 mg/mL. To pinpoint the most effective formulation, a desirability analysis was conducted, which predicted a superior optimal MIC of 0.21 mg/mL (Figure 4D). This peak response, associated with a high desirability score of 0.99, is obtained with a specific binary mixture containing 15% Immortelle and 85% Oregano by volume.

### 3.7. Simultaneous Optimization of All Responses

To identify a single essential oil formulation capable of effectively inhibiting all four bacterial strains concurrently, a simultaneous optimization was performed using the desirability function approach. This statistical tool assesses how closely a set of responses aligns with the defined optimal goals, integrating them into a single composite score known as the overall desirability. The objective was to find the specific blend of Immortelle, Parsley, and Oregano that maximizes this overall desirability, thereby representing the best possible compromise for inhibiting all tested pathogens simultaneously.

**Figure 5:**
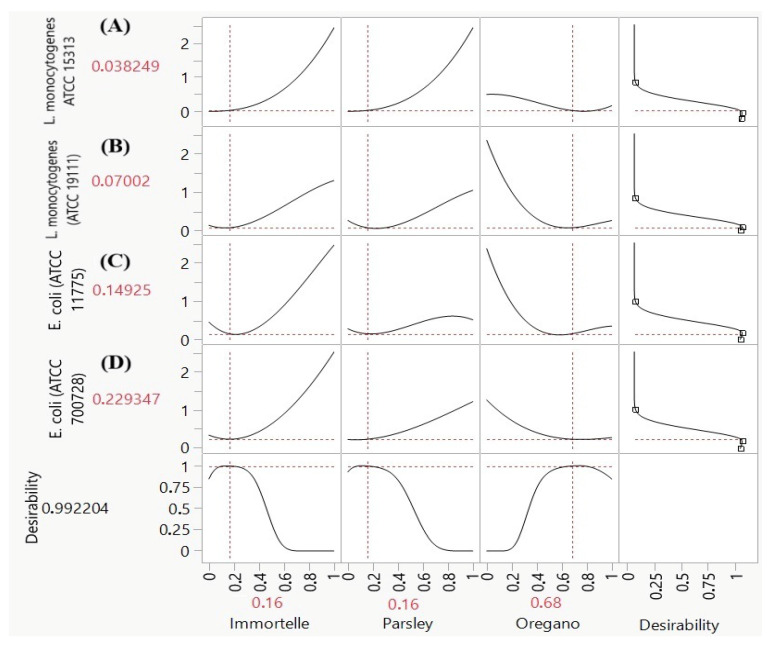
Desirability plots of the simultaneous optimization of MIC LM1 (**A**), MIC LM2 (**B**), MIC EC1 (**C**) and MIC EC2 (**D**).

This analysis successfully identified an optimal formulation that achieved a near-perfect overall desirability score of 0.9922 (Figure 5), indicating its high efficacy against all four bacterial strains concurrently. The ideal blend consists of a ternary mixture composed of 16% Immortelle, 16% Parsley, and 68% Oregano (*v*/*v*/*v*). At this specific ratio, the model predicted the following Minimum Inhibitory Concentration (MIC) values: 0.038 mg/mL for *L. monocytogenes* (ATCC 15313), 0.070 mg/mL for *L. monocytogenes* (ATCC 19111), 0.149 mg/mL for *E. coli* (ATCC 11775), and 0.229 mg/mL for *E. coli* (ATCC 700728). Therefore, this specific formulation, which is notably dominated by Oregano, represents a statistically optimized solution for simultaneously inhibiting the growth of all tested pathogenic strains, confirming its potential as an effective broad-spectrum antimicrobial agent.

### 3.8. Experimental Verification of the Assumed Model

The reliability of the special cubic models for the five responses, MIC LM1, MIC LM2, MIC EC1, and MIC EC2, was verified through a validation test. This assessment compared the predicted values with the corresponding experimental results. The selected test points reflect the essential oil proportions obtained from the simultaneous optimization of all of the tested responses.

As shown in Table 5, the experimental data exhibit a strong correlation with the predicted values, and no statistically significant differences were observed between them. These findings confirm the accuracy and suitability of the proposed and validated models.

### 3.9. Synergy Between EOs

Interactions among different essential oils, as well as between their primary and secondary constituents, can result in additive, synergistic, or even antagonistic effects. Because pathogens are unlikely to develop resistance to the numerous bioactive components present in two or more essential oils, combining them can be particularly beneficial. In such mixtures, synergistic interactions among different oils—or among the individual compounds within multiple extracts—can enhance their antibacterial activities [54].

The mixture design analysis performed in this study successfully identified an optimal formulation indicating high concurrent efficacy against all four tested bacterial strains. The ideal blend was determined to be a ternary mixture composed of 16% *Helichrysum plicatum*, 16% *P. crispum*, and 68% *O. vulgare* (*v*/*v*/*v*). These findings confirm that the optimized formulation serves as an effective broad-spectrum antimicrobial agent, a result that aligns with the established view that essential oil mixtures can exhibit enhanced activity through synergistic interactions [55].

The enhanced antibacterial activity observed in the combined application of *P. crispum*, *Helichrysum plicatum*, and *O. vulgare* essential oils suggests a potent synergy among their volatile compounds. This finding aligns with established principles in natural product research, where the antimicrobial efficacy of complex essential oil (EO) mixtures often exceeds the sum of their individual components due to multi-target interactions [55,56]. The synergy recorded in this study can be rationalized by analyzing the specific roles of the major compounds present in these oils: carvacrol, *p*-cymene, α-pinene, 1,3,8-p-menthatriene, and γ-curcumene.

The dominance of *O. vulgare* (68%) in the optimized blend suggests it acts as the primary driver of antimicrobial toxicity. Origanum is rich in potent bioactive compounds such as carvacrol, thymol, α-pinene, and *p*-cymene, which are well-documented for their significant antimicrobial activity [57]. Previous studies utilizing mixture-design methodologies have consistently identified carvacrol-rich *Origanum* oils as the dominant active component in synergistic blends, effective against a range of Gram-positive and Gram-negative pathogens including *Staphylococcus aureus* and *E. coli* [58]. Carvacrol, in particular, exhibits strong antibacterial effects [59]. However, the inclusion of *P. crispum* and *Helichrysum plicatum* at 16% each is critical to the observed efficacy, likely facilitating a synergistic response where the combined effect surpasses that of the individual oils. This supports the concept that minor or less active constituents can potentiate the activity of major compounds. For instance, while *p*-cymene is often inactive on its own, it has been shown to produce significant synergistic effects when combined with other terpenes like carvacrol or 1,8-cineole [60].

The contribution of *P. crispum* to this synergy is particularly notable given its chemical profile, which includes 1,3,8-p-menthatriene. This observation is reinforced by recent findings from Kobacy et al., who demonstrated that while *P. crispum* may exhibit lower standalone inhibition zones compared to membrane-disrupting oils, it acts as a vital potentiator in mixtures [61]. Specifically, Kobacy et al., suggest that while volatile phenols disrupt the bacterial cell membrane [61], *P. crispum* constituents interfere with intracellular metabolic pathways. This distinct mechanism facilitates a multi-targeted synergistic attack, broadening the antimicrobial spectrum and amplifying the overall efficacy of the formulation beyond what is achievable by single oils [61]. Similarly, the inclusion of *Helichrysum plicatum* aligns with reports that medicinal plants can possess combined synergistic effects [62]. By optimizing the ratio of these oils, the formulation likely leverages interactions between “inactive” precursors and potent agents like carvacrol and α-pinene, optimizing the disruption of bacterial pathogens [59]. The robust activity observed in our study supports the concept that multicomponent mixtures can offer superior therapeutic potential compared to single isolates, likely by attacking multiple cellular targets simultaneously and reducing the potential for resistance [56]. Also, this study confirms that a specific, Oregano-dominated ternary blend offers a statistically optimized solution for inhibiting both Gram-positive and Gram-negative bacteria, offering a promising natural alternative to conventional antibiotics.

### 3.10. Drug-Likeness and ADME-Toxicity Predictions

The pharmacokinetic features of absorption, distribution, metabolism, excretion, and toxicity (ADMET) of four main compounds were predicted based on their physicochemical properties. The results demonstrated that all five Lipinski’s rules were well satisfied, with molecular weights below 500 g/mol, lipophilicity (Log P) values under 5, molar refractive index between 40 and 130, hydrogen bond acceptors less than ten, and hydrogen bond donors not exceeding five, as shown in Table 6.

Furthermore, the main compounds were predicted to have high human intestinal absorptions (HIAs > 95%), good levels of permeability to the central nervous system and the blood–brain barrier, and strong inhibition of cytochrome P450 1A2. They were predicted to be safe inhibitors with negative AMES tests for genetic transformation, negative side effects on the liver, and skin sensitivity issues on the human body, except for γ-curcumene (M2), 1,3,8-p-menthatriene (M3), and carvacrol (M4), which were predicted to have positive skin sensitization, as presented in Table 7.

**Figure 6:**
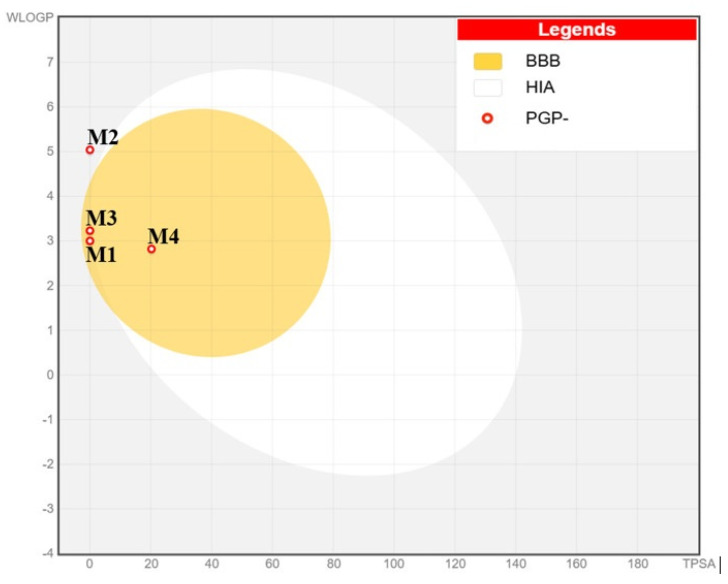
Absorption in the gastrointestinal tract and BBB penetration based on the boiled egg model for M1: α-pinene, M2: γ-curcumene, M3: 1,3,8-p-menthatriene, M4: carvacrol.

The predictive model, known as BOILED-Egg, combines lipophilicity (WLOGP) and topological polar surface area (TPSA) to predict two key pharmacokinetic behaviors: negative absorption in the gastrointestinal tract, as defined by the white area, and blood–brain barrier penetration referred to by the yellow area. As a result, α-pinene (M1), 1,3,8-p-menthatriene (M3), and carvacrol (M4) are part of the yellow Egan egg, revealing their promising role to penetrate the blood–brain barrier as presented in Figure 6.

**Figure 7:**
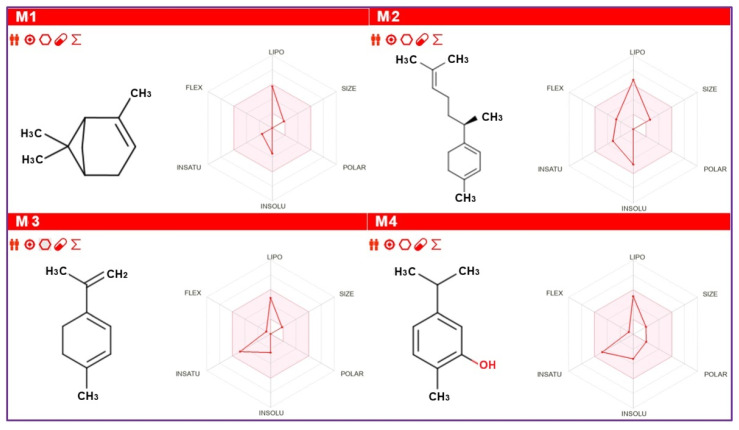
Bioavailability radars for four major compounds for M1: α-pinene, M2: γ-curcumene, M3: 1,3,8-p-menthatriene, M4: carvacrol. The Pink Shaded Area represents the physicochemical space for optimal oral bioavailability. The Red Line (Compound Profile) represents the specific calculated properties of the molecule shown (M1, M2, M3, M4).

Furthermore, the bioavailability radars demonstrate excellent bioavailability predictions for all major compounds because they meet the six physicochemical features, including lipophilicity, saturation, polarity, flexibility, solubility, and size, as represented by the pink area. This pink zone defines the ideal physicochemical space for oral bioavailability, as illustrated in Figure 7.

### 3.11. Molecular Docking Simulations

The compounds under study were equally examined through a molecular docking analysis to identify their inhibition mechanisms towards two responsible receptors, targeting *E. coli* and *L. monocytogenes* strains, encoded by 6F86 and 5ZQB, respectively.

**Figure 8:**
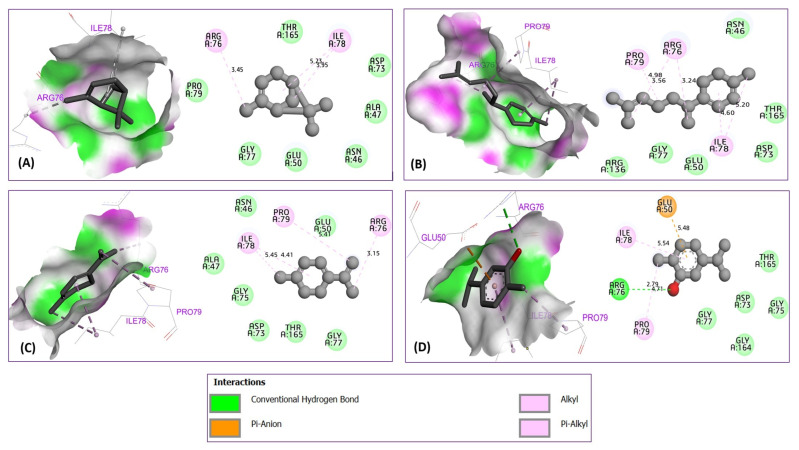
Intermolecular interactions in three and two-dimensional views for α-pinene (**A**), γ-curcumene (**B**), 1,3,8-p-menthatriene (**C**), and carvacrol (**D**) in complex with 6F86 protein from *E. coli*.

**Figure 9:**
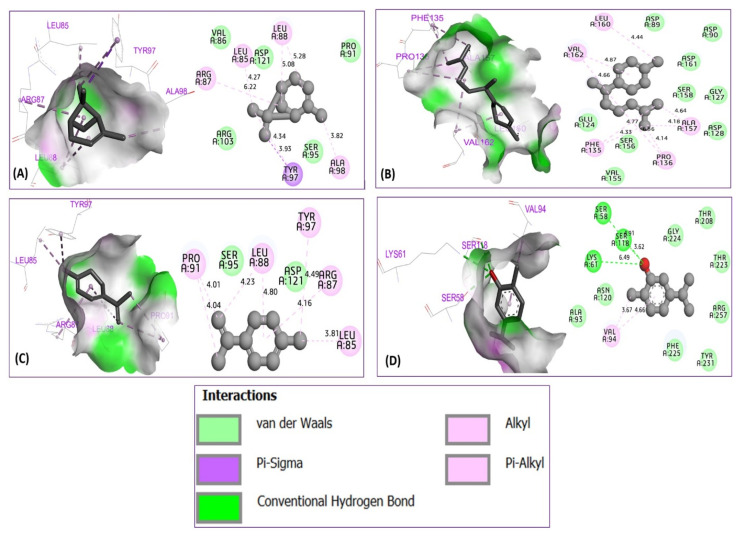
Intermolecular interactions in three and two-dimensional views for α-pinene (**A**), γ-curcumene (**B**), 1,3,8-p-menthatriene (**C**), and carvacrol (**D**) in complex with 6F86 protein from *L. monocytogenes*.

The obtained results demonstrate that α-pinene was complexed to 6F86 with a binding energy of −5.23 kcal/mol, producing two alkyl bonds detected with Arg76 and Ile78 amino acid residues. γ-curcumene and 1,3,8-p-menthatriene were docked to the antibacterial protein with binding energies of −5.40 and −5.02 kcal/mol, respectively, revealing three common alkyl bonds which were formed with Arg67, Ile78, and Pro79 residues, so that carvacrol (binding energy of −5.25 kcal/mol) revealed one conventional hydrogen bond with Arg76 residue, one Alkyl bond with Pro79, one Pi-Alkyl bond with Ile78, and a Pi-anion bond with Glu50 amino acid residue, as presented in Figure 8. The same compounds were docked to the antibacterial protein from *L. monocytogenes* strains (PDB ID of 5ZQB), producing one Pi-Sigma bond with Tyr97, four Alkyl bonds with Ala98, Leu88, Leu85, and Arg87 for α-pinene (binding energy of −5.61 kcal/mol). While γ-curcumene (binding energy of −5.36 kcal/mol) was docked to the second targeted protein, revealing five Alkyl bonds detected towards Leu60, Val162, Ala157, Phe135, and Pro136 residues. 1,3,8-p-menthatriene complexed to 5ZQB protein with a binding energy of −5.42 kcal/mol, revealed five Alkyl bonds detected with Pro91, Leu88, Tyr97, Arg87, and Leu85 residues. For carvacrol complexed with a binding energy of −5.01 kcal/mol, three conventional hydrogen bonds were detected towards Ser58, Ser118, and Lys61, more than one Alkyl bond created with Val94 amino acid residue, as shown in Figure 9.

In conclusion, the molecular docking findings highlight various interaction modes of these extracted molecules, making them promising natural candidates with strong inhibitory potential through the formation of stable complexes with key residues in 6F86 and 5ZQB for the development of targeted antibacterial agents. Future studies should focus on experimental validation of the in silico findings, including cytotoxicity assays on human normal cell lines to confirm biocompatibility, as well as scanning electron microscopy (SEM) analysis to elucidate the antibacterial mechanism by assessing bacterial morphological alterations and cell membrane damage.

## 4. Conclusions

This study demonstrated that a ternary mixture of *H. plicatum*, *P. crispum*, and *O. vulgare* essential oils exhibits enhanced antibacterial activity against *Listeria monocytogenes* and *Escherichia coli* strains isolated from milk, with oregano oil playing a dominant role in the optimized formulation. The augmented simplex-centroid mixture design identified a specific ratio (16:16:68, *v*/*v*/*v*) as the most effective combination, highlighting the importance of synergistic interactions among the essential oils. In silico ADMET and molecular docking analyses further supported these findings by indicating favorable pharmacokinetic properties, acceptable safety profiles, and stable interactions of major oil constituents with key bacterial targets. Together, the experimental and computational results provide a mechanistic and quantitative basis for the rational design of essential oil-based antimicrobial formulations. These findings support the potential application of optimized essential oil mixtures as natural preservatives in dairy products, while future studies should focus on experimental validation of safety and antibacterial mechanisms.

## Figures and Tables

**Table 1:** Chemical composition of *P. crispum*, *H. plicatum* and *O. vulgare* EOs.

#	Compound ^a^	RI_exp._^b^	RI_lit._^c^	Immortelle ^d^	Parsley	Oregano
				%m/m	%m/m	%m/m
1	4-trans-octene	800.8	801	0.11	-	-
2	4-methyl-3-hexanone *	839.7	804	0.12	-	-
3	α-thujene	926.6	924	-	0.14	1.18
4	α-pinene	936.0	932	27.61	17.34	0.92
5	thuja-2,4(10)-diene	946.6	953	-	-	0.06
6	Camphene	950.8	946	0.44	0.11	0.27
7	1-octen-3-ol	976.8	974	-	-	0.88
8	Sabinene	975.5	969	-	0.74	-
9	β-pinene	981.4	974	0.50	12.40	0.20
10	3-octanone	984.6	979	-	-	0.27
11	Myrcene	990.3	988	0.10	5.14	1.82
12	3-octanol	993.2	988	-	-	0.06
13	dehydro-1,8-cineole	995.4	988	-	0.01	-
14	α-phellandrene	1007.6	1002	-	0.71	0.17
15	δ-3-carene	1013.2	1008	-	-	0.09
16	α-terpinene	1019.6	1014	0.21	0.04	1.06
17	*p*-cymene	1027.6	1022	0.24	0.12	15.38
18	Sylvestrene	1031.8	1025	3.26	-	0.28
19	β-phellandrene	1034.6	1025	-	10.41	0.20
20	1,8-cineole	1035.8	1026	0.47	-	0.12
21	*cis*-β-ocimene	1047.2	1032	-	-	0.06
22	*trans*-β-ocimene	1048.5	1044	-	0.09	-
23	isobutyl angelate	1049.9	1045	0.20	-	-
24	g-terpinene	1061.8	1054	0.57	0.49	5.15
25	n.i.	1070.8		-	-	0.36
26	n.i.	1077.8		-	-	0.02
27	Terpinolene	1092.4	1086	0.23	4.60	0.17
28	Linalool	1100.1	1095	0.92	0.02	0.23
29	n.i.	1102.5		-	-	0.22
30	2-methyl butyl-2-methyl butyrate	1102.9	1100	0.13	-	-
31	Perillene	1103.7	1102	-	0.01	-
32	*endo*-fenchol	1119.2	1114	0.05	-	-
33	1,3,8-*p*-menthatriene	1120.8	1108	-	23.66	-
34	*trans*-thujone	1121.7	1112	-	-	0.07
35	1-terpineol	1126.5	1130	-	-	0.04
36	menthatriene isomer (unknown)	1143.4		-	0.38	-
37	isoamyl tiglate	1152.3	1148	0.61	-	-
38	nerol oxide	1156.5	1154	0.06	-	-
39	camphor	1153.3	1141	-	-	0.04
40	*iso*-menthone	1159.9	1158	-	-	0.04
41	pentyl cyclohexa-1,3-diene	1163.6	1156	-	0.03	-
42	borneol	1173.7	1165	0.06	-	0.58
43	*iso*-menthol	1180.6	1179	-	0.02	-
44	terpinen-4-ol	1184.4	1174	0.29	0.05	0.82
45	n.i. (mw = 170)	1188.3		0.14	-	-
46	*p*-cymen-8-ol	1190.0	1179	-	0.09	0.14
47	2-decanone	1191.6	1190	0.05	-	-
48	g-terpineol	1197.1	1199	0.47	0.04	0.17
49	*cis*-dihydro carvone	1203.0	1191	-	-	0.05
50	*n*-decanal	1204.6	1201	0.12	-	-
51	*trans*-dihydro carvone	1205.4	1200	-	-	0.18
52	myrtenal	1206.3	1195	-	0.11	-
53	*o*-cumenol	1211.9	1196	-	0.03	-
54	*cis*-ocimenone	1229.1	1226	-	0.06	-
55	nerol	1229.8	1227	0.59	-	-
56	hexyl 2-methyl butanoate	1235.6	1233	0.05	-	-
57	carvacrol methyl ether	1246.3	1241	-	-	0.46
58	*trans*-ocimenone	1252.8	1235	-	0.05	-
59	carvone	1256.5	1239	-	-	0.07
60	2-phenyl ethyl acetate	1259.6	1254	0.04	-	-
61	*p*-menth-1-en-7-al	1277.4	1273	-	0.01	-
62	neryl formate	1281.2	1280	0.23	-	-
63	*p*-cymen-7-ol	1284.2	1289	-	-	0.04
64	thymol	1291.2	1289	0.05	-	6.05
65	2-undecanone	1292.6	1293	0.04	-	-
66	bornyl acetate	1295.0	1284	-	0.02	-
67	tridecane	1298.0	1300	0.04	0.01	-
68	carvacrol	1308.3	1298	-	-	58.29
69	δ-terpinyl acetate	1318.9	1316	-	-	0.02
70	*trans*-patchenol	1328.6	1328	-	0.05	-
71	n.i.	1332.3		-	-	0.08
72	citronellyl acetate	1346.7	1350	-	0.01	-
73	neryl acetate	1364.2	1359	5.88	-	-
74	β-panasinsene	1382.2	1381	0.27	-	-
75	α-ylangene	1385.1	1373	0.15	-	-
76	β-cubebene	1388.5	1387	-	0.02	-
77	α-copaene	1389.9	1374	1.61	-	-
78	α-duprezianene	1391.8	1387	0.57	-	-
79	daucene	1392.2	1380	-	0.04	-
80	*cis*-caryophyllene	1401.7	1408	-	0.09	-
81	*cis*-β-farnesene	1416.3	1440	-	0.05	-
82	β-duprezianene	1421.4	1421	2.74	-	-
83	α-*cis*-bergamotene	1424.4	1411	1.18	0.05	-
84	g-elemene	1438.4	1434	-	-	1.94
85	aromadendrene	1439.0	1439	4.89	-	-
86	*trans*-β-farnesene	1440.3	1454	-	0.06	-
87	α-*trans*-bergamotene	1445.8	1432	2.16	-	-
88	n.i.	1446.9		-	-	0.13
89	neryl propanoate	1452.9	1452	1.87	-	-
90	*cis*-cadina-1(6),4-diene	1457.6	1461	1.04	-	-
91	α-acoradiene	1465.4	1464	0.21	-	-
92	α-humulene	1472.7	1470	-	-	0.20
93	cumacrene	1473.0	1470	0.39	-	-
94	*trans*-muurola-4(14),5-diene	1479.6	1493	-	0.03	-
95	β-acoradiene	1481.8	1469	0.33	-	-
96	α-amorphene	1484.4	1483	0.23	-	-
97	γ-curcumene	1490.2	1481	20.71	-	-
98	*ar*-curcumene	1491.9	1479	1.73	-	-
99	Italidione II (mw = 224)	1495.8		1.20	-	-
100	germacrene D	1500.6	1484	-	0.09	-
101	α-zingiberene	1502.7	1493	0.27	-	-
102	β-bisabolene	1507.7	1505	6.10	-	-
103	*cis*-dihydroagarofuran	1510.4	1519	-	0.10	-
104	7-*epi*-α-selinene	1515.6	1520	3.97	-	-
105	β-sesquiphellandrene	1516.4	1521	-	-	0.80
106	β-curcumene	1519.4	1514	0.45	-	-
107	*cis*-γ-bisabolene	1521.0	1514	-	0.01	-
108	δ-cadinene	1522.5	1522	0.22	-	-
109	*trans*-γ-bisabolene	1526.8	1529	0.07	-	-
110	*trans*-cadina-1,4-diene	1530.3	1533	-	-	0.04
111	zonarene	1530.9	1528	0.27	-	-
112	italicene ether	1534.2	1536	0.08	-	-
113	α-cadinene	1536.4	1537	-	-	0.08
114	myristicin	1538.6	1517	-	12.72	-
115	α-cadinene	1537.1	1537	0.84	-	-
116	selina-3,7(11)-diene	1541.8	1545	0.16	-	-
117	n.i.	1549.5		0.16	-	-
118	kessane *	1552.9	1529	-	0.10	-
119	Italicene ether (unknown isomer)	1553.6		0.13	-	-
120	thymohydroquinone	1553.8	1553	-	-	0.05
121	β-calacorene	1560.7	1564	0.06	-	-
122	elemicin	1561.5	1555	-	3.41	-
123	*trans*-nerolidol	1567.4	1561	0.09	-	-
124	neryl isovalerate	1583.3	1582	0.20	-	-
125	n.i.	1585.3		-	0.04	-
126	1-hexadecene	1588.4	1588	0.67	-	-
127	1-allyl-2,3,4,5-tetramethoxybenzene	1606.0	1591	-	4.18	-
128	humulene epoxide II	1607.5	1608	-	-	0.27
129	*cis*-sesquilavandulol	1607.8	1607	0.07	-	-
130	guaiol	1614.8	1600	0.11	-	-
131	n.i.	1920.4		-	-	0.08
132	1-*epi*-cubenol	1628.7	1627	0.13	-	-
133	rosifoliol *	1631.9	1600	0.19	-	-
134	hinesol	1636.1	1640	0.09	-	-
135	carotol *	1636.4	1594	-	0.23	-
136	n.i.	1947.9		-	-	0.09
137	cubenol	1653.6	1645	0.06	-	-
138	β-eudesmol	1655.8	1649	0.05	-	-
139	*cis*-nerolidyl acetate	1674.4	1676	0.07	-	-
140	elemol acetate	1678.6	1680	0.15	-	-
141	*epi*-α-bisabolol	1681.9	1683	0.08	-	-
142	n.i.	1695.4		0.05	-	-
143	apiole	1695.5	1677	-	1.58	-
144	n.i.	1729.2		0.07	-	-
145	n.i.	2008.2		-	0.14	-
146	n.i.	2091.7		-	0.04	-
	SUM of identified			99.58	99.78	99.03
	Monoterpene hydrocarbons			33.15	76.38	27.01
	Oxigenated monoterpene hydrocarbons			9.99	0.54	67.44
Total monoterpene hydrocarbons				43.14	76.92	94.45
	Sesquiterpene hydrocarbons			50.65	0.45	3.32
	Oxigenated sesquiterpene hydrocarbons			3.35	0.48	-
Total sesquiterpene hydrocarbons				54.00	0.92	3.32
	Italidiones			1.20	-	-
	Phenylpropanoids			-	21.89	-
	Aliphatic			0.82	0.01	-
	Aliphatic oxygenated			0.38	-	1.21
	Other			0.46	0.25	1.02

^a^ n.i.—stands for not identified compounds inside Table 1. ^b^ RI exp.—experimental retention indices calculated relative to a homologous series of n-alkanes (C7–C40) on an InertCap-5 column. ^c^ RI lit.—literature retention indices reported in the Adams [13], and NIST [11] databases. ^d^ GC analyses were performed without experimental replicates due to the use of commercially available essential oils assumed to be homogeneous mixtures. * Tentative identification; mass spectra show a high degree of similarity, but the difference between RI exp. and RI lit. exceeds 20 units.

**Table 2:** Experimental conditions of mixture design along with actual and predicted of each experiment for antibacterial activities (mg mL^−1^).

Exp N ^a^	PC	HP	OV	*L. monocytogenes* *(ATCC 15313)*		*L. monocytogenes* *(ATCC 19111)*		*E. coli* (ATCC 11775)		*E. coli O157:H7* (ATCC 700728)	
				Actual	Predicted	Actual	Predicted	Actual	Predicted	Actual	Predicted
1	1	0	0	2.50	2.44	1.25	1.31	2.50	2.47	2.50	2.53
2	0	1	0	2.50	2.44	1.25	1.05	0.63	0.51	1.25	1.22
3	0	0	1	0.08	0.18	0.16	0.26	0.31	0.35	0.31	0.27
4	0.5	0.5	0	0.63	0.50	2.50	2.35	2.50	2.35	1.25	1.25
5	0.5	0	0.5	0.31	0.34	0.31	0.46	0.63	0.64	0.63	0.62
6	0	0.5	0.5	0.31	0.34	0.31	0.21	0.63	0.55	0.63	0.56
7	0.33	0.33	0.33	0.63	0.34	0.63	0.48	0.31	0.42	0.31	0.48
8	0.33	0.33	0.33	0.16	0.34	0.31	0.48	0.63	0.42	0.63	0.48
9	0.33	0.33	0.33	0.31	0.34	0.63	0.48	0.63	0.42	0.63	0.48
10	0.67	0.17	0.17	0.63	0.85	1.25	1.13	1.25	1.45	1.25	1.21
11	0.17	0.67	0.17	0.63	0.85	0.31	0.96	0.31	0.77	0.63	0.76
12	0.17	0.17	0.67	0.31	0.04	0.31	0.07	0.16	0.14	0.08	0.23
E211				2.00	-	1.00	-	2.00	-	1.00	-

^a^: Experiments were carried out after randomization.

**Table 3:** Variance analysis for the fitted models by MDM.

MIC *L. monocytogenes* (ATCC 15313)	Model	DF	SS	MS	F	*p*-value
	R	6	7.43	1.24	19.27	0.0026 *
	r	5	0.32	0.06		
	LOF	3	0.206	0.069	1.193	0.4862
	PE	2	0.115	0.058		
	Total	11		7.754		
	R^2^	0.96				
	R^2^_adj_	0.91				
MIC *L. monocytogenes* (ATCC 19111)	Model	DF	SS	MS	F	*p*-value
	R	6	4.47	0.75	5.48	0.04 *
	r	5	0.68	0.14		
	LOF	3	0.61	0.20	5.97	0.15
	PE	2	0.07	0.03		
	Total	11	5.15			
	R^2^	0.87				
	R^2^_adj_	0.71				
MIC *E. coli* (ATCC 11775)	Model	DF	SS	MS	F	*p*-value
	R	6	6.79	1.13	14.26	0.005 *
	r	5	0.40	0.08		
	LOF	3	0.33	0.11	3.21	0.25
	PE	2	0.07	0.03		
	Total	11	7.19			
	R^2^	0.94				
	R^2^_adj_	0.88				
MIC *E. coli* (ATCC 700728)	Model	DF	SS	MS	F	*p*-value
	R	6	4.50	0.75	30.69	0.0009 *
	r	5	0.12	0.02		
	LOF	3	0.05	0.02	0.53	0.7072
	PE	2	0.07	0.03		
	Total	11	4.62			
	R^2^	0.97				
	R^2^_adj_	0.94				

R: Regression; r: residual; DF: degrees of freedom; SS: sum of squares; MS: mean square; F: Calculated Fisher value; R^2^: Coefficient of determination; adj: Adjusted; LOF: lack of fit; PE: Pure Error; *: statistically significant at 95% confidence level.

**Table 4:** Estimated regression coefficients of the special cubic model.

Term	Coefficient	MIC*_LM (ATCC 15313)_*		MIC*_LM(ATCC 19111)_*		MIC*_E. coli (ATCC 11775)_*		MIC*_LM E. coli (ATCC 700728)_*	
		Estimate	*p*-Value	Estimate	*p*-Value	Estimate	*p*-Value	Estimate	*p*-Value
Immortelle	β_1_	2.44	0.0002 *	1.31	0.0145 *	2.47	0.0003 *	2.53	<0.0001 *
Parsley	β_2_	2.44	0.0002 *	1.05	0.032 *	0.51	0.1182	1.22	0.0005 *
Oregano	β_3_	0.18	0.4996	0.26	0.5022	0.35	0.2536	0.27	0.1308
Immortelle × Parsley	β_12_	−7.73	0.0015 *	4.71	0.0467 *	3.44	0.0536	−2.51	0.0215 *
Immortelle × Oregano	β_13_	−3.85	0.0262 *	−1.27	0.5091	−3.08	0.0746	−3.12	0.0093 *
Parsley × Oregano	β_23_	−3.85	0.0262 *	−1.79	0.3648	0.49	0.7365	−0.73	0.3788
Immortelle × Parsley × Oregano	β_123_	9.96	0.1976	−15.62	0.1703	−21.31	0.0355 *	−4.05	0.3719

*: statistically significant at 95% confidence level.

**Table 5:** Predicted and experimental values for the test point realized for the optimal mixtures.

Responses	MDM	
	Predicted ^a^	Experimental ^b^
MIC*_LM1_* (mg/mL)	0.034	0.03 ± 0.00
MIC*_LM2_* (mg/mL)	0.048	0.05 ± 0.00
MIC_EC1_ (mg/mL)	0.12	0.15 ± 0.00
MIC_EC2_ (mg/mL)	0.21	0.20 ± 0.00

^a^: Predicted values calculated using the model under the determined optimal formulation. ^b^: Experimental values expressed as mean ± standard deviation (n = 3).

**Table 6:** Physicochemical features of four major compounds M1: α-pinene, M2: γ-curcumene, M3: 1,3,8-p-menthatriene, M4: carvacrol.

Compounds Number	Physico-Chemical Properties					Lipinski’s Five Rules
	MW	MR Index	Log P	HBA	HBD	(No/Yes)
Rule	≤500 (g/mol)	130 ≥ MR Index ≥ 40	<5	≤10	<5	
M1	136.23	45.22	2.63	0	0	Yes
M2	204.35	70.68	3.57	0	0	Yes
M3	134.22	46.65	2.58	0	0	Yes
M4	150.22	48.01	2.24	1	1	Yes

**Table 7:** ADMET pharmacokinetic features of four major compounds M1:α-pinene, M2: γ-curcumene, M3: 1,3,8-p-menthatriene, M4: carvacrol.

Compounds Number	A	D		M							E	Tox		
	Human Intestinal Absorption	Blood–Brain Barrier Permeability	Central Nervous System Permeability	Substrate		Inhibitor					Total Clearance	AMES Test of Toxicity	Hepatotoxicity	Skin Sensitization
				Cytochromes										
				2D6	3A4	1A2	2C19	2C9	2D6	3A4				
	(% Absorbed)	(Log BB)	(Log PS)	(No/Yes)							Numeric (Log ml/min/kg)	(No/Yes)		
M1	98.77	0.531	−1.84	No	No	Yes	No	No	No	No	0.053	No	No	No
M2	96.489	0.661	−1.70	No	No	Yes	No	No	No	No	1.24	No	No	Yes
M3	98.195	0.563	−2.006	No	No	Yes	No	No	No	No	0.263	No	No	Yes
M4	95.56	0.355	−1.168	No	No	Yes	No	No	No	No	0.23	No	No	Yes

## Data Availability

The original contributions presented in this study are included in the article. Further inquiries can be directed to the corresponding author.
